# Supplementary material for: Using Front-Face Fluorescence Spectroscopy and Biochemical Analysis of Honey to Assess a Marker for the Level of Varroa destructor Infestation of Honey Bee (Apis mellifera) Colonies
Source: Foods. 2023 Feb 2;12(3):629. doi: 10.3390/foods12030629 (PMC9914405; doi:10.3390/foods12030629)
Supplement: Supplementary file 1 [file foods-12-00629-s001.zip › foods-2038026-supplementary.pdf]

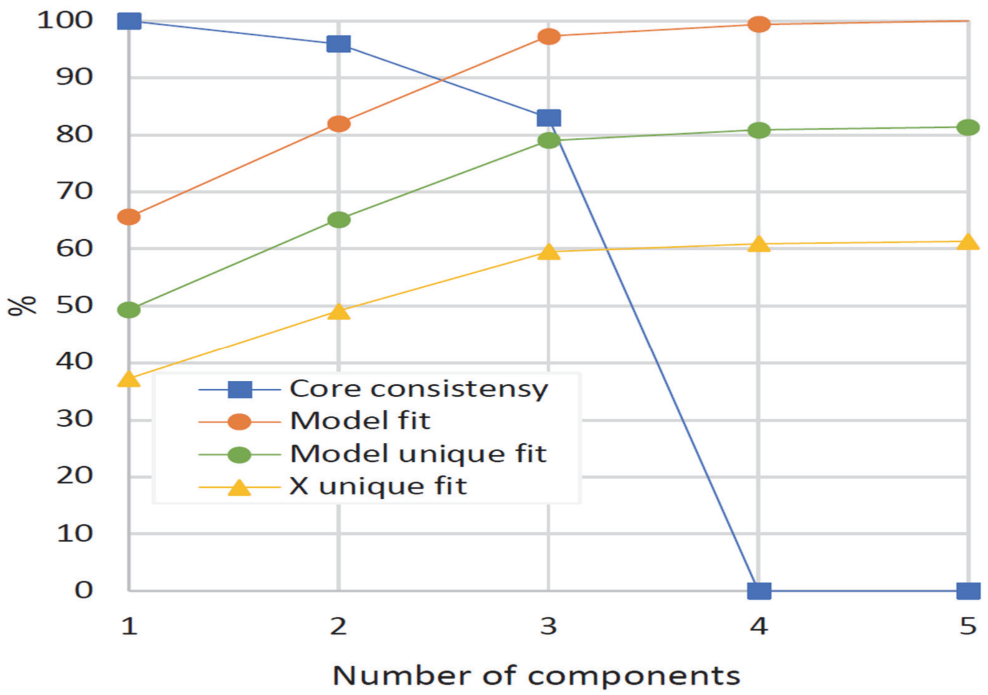

Figure S1. Core consistency and percent of explained variance expressed as model fit, model unique fit and unique fit of X, for five PARAFAC models with number of components increasing from 1 to 5.

Table S1. Concentrations of different pollen types which dominate in honey samples from the beehives of different infestation level with *V. destructor*

	Pollen concentration (PG/10 g)									
Infestation (%)	0	0	0	0.054	0.056	0.058	0.10	0.26	0.62	1.25
Ambrosia sp.				89.32						
Amorpha fruticosa	45.56	56.44	11.76		25.45		48.66	20.95	77.59	
Gleditsia triacanthos			17.24			21.77				
Prunus sp.						43.08		17.14		33.53
Robinia pseudoacacia								18.09		
Trizonocolporate reticulate			12.58							
unidentifiable	16.67				32.30	15.87	23.95	28.09		
Vitis sp.										28.29
